# Comparative Virus-Host Protein Interactions of the Bluetongue Virus NS4 Virulence Factor

**DOI:** 10.3390/v14020182

**Published:** 2022-01-19

**Authors:** Aurore Fablet, Cindy Kundlacz, Juliette Dupré, Edouard Hirchaud, Lydie Postic, Corinne Sailleau, Emmanuel Bréard, Stéphan Zientara, Damien Vitour, Grégory Caignard

**Affiliations:** 1 UMR VIROLOGIE, INRAE, École Nationale Vétérinaire d’Alfort, ANSES Laboratoire de Santé Animale, Université Paris-Est, 94700 Maisons-Alfort, France; aurore.fablet@vet-alfort.fr (A.F.); cindy.kundlacz@free.fr (C.K.); juliette.dupre@vet-alfort.fr (J.D.); lydie.postic@anses.fr (L.P.); corinne.sailleau@anses.fr (C.S.); emmanuel.breard@anses.fr (E.B.); stephan.zientara@anses.fr (S.Z.); 2 IGFL, CNRS UMR5242, ENS Lyon, 69007 Lyon, France; 3 Ploufragan-Plouzané-Niort Laboratory, Viral Genetic and Biosecurity Unit, ANSES, 22440 Ploufragan, France; edouard.hirchaud@anses.fr

**Keywords:** Bluetongue virus, NS4, WTAP, protein–protein interaction

## Abstract

Bluetongue virus (BTV) is the etiologic agent of a non-contagious arthropod-borne disease transmitted to wild and domestic ruminants. BTV induces a large panel of clinical manifestations ranging from asymptomatic infection to lethal hemorrhagic fever. Despite the fact that BTV has been studied extensively, we still have little understanding of the molecular determinants of BTV virulence. In our report, we have performed a comparative yeast two-hybrid (Y2H) screening approach to search direct cellular targets of the NS4 virulence factor encoded by two different serotypes of BTV: BTV8 and BTV27. This led to identifying Wilms’ tumor 1-associated protein (WTAP) as a new interactor of the BTV-NS4. In contrast to BTV8, 1, 4 and 25, NS4 proteins from BTV27 and BTV30 are unable to interact with WTAP. This interaction with WTAP is carried by a peptide of 34 amino acids (NS4^22−55^) within its putative coil-coiled structure. Most importantly, we showed that binding to WTAP is restored with a chimeric protein where BTV27-NS4 is substituted by BTV8-NS4 in the region encompassing residue 22 to 55. We also demonstrated that WTAP silencing reduces viral titers and the expression of viral proteins, suggesting that BTV-NS4 targets a cellular function of WTAP to increase its viral replication.

## 1. Introduction

Bluetongue virus (BTV) is the etiological agent of the Bluetongue disease, a non-contagious arbovirus that affects a wide range of wild and domestic ruminants. It is transmitted by blood-feeding midges of the genus *Culicoides*. Up to 2008, 24 serotypes had been principally identified by a virus neutralization test. This group of serotypes, named as classical BTV serotypes (BTV1–24), can infect a broad spectrum of ruminants (especially sheep) and the disease is associated with ulcer in the oral cavity and upper gastrointestinal tract, hemorrhagic fever, necrosis of the skeletal and cardiac muscle and oedema of the lungs [1]. Since 2008, progress in molecular diagnosis and high-throughput sequencing approach (next generation sequencing) have led to the discovery of an increased number of new strains within serotypes but also new serotypes of BTV. These newly identified BTV-serotypes are characterized by a low (sometimes even an absence of) pathology/virulence, their capacity to infect only small ruminants such as goats and, for some of them, their direct transmission [2,3]. Today, 36 serotypes have been identified; the serotypes 25 to 36 are called small ruminants adapted serotypes, non-transmitted by *Culicoides* [4]. Consequently, this non-classical group of BTV serotypes (BTV25-36) is also named “atypical” BTVs, distinct from the classical BTV1–24.

The Bluetongue virus particle is icosahedral and organized as a triple-layered capsid that incorporates 10 double-stranded RNA (dsRNA) segments encoding seven structural (VP1 to VP7) and five, or possibly six, non-structural (NS1 to NS4, NS3A and possibly NS5) proteins [5,6,7]. All these viral genomic segments are associated with the replication complex, which is composed of VP1 (RNA-dependent RNA polymerase), VP4 (capping enzyme including methyltransferase), VP6 (RNA-dependent ATPase and helicase) and enclosed by VP3 (subcore) and VP7 (core) [8]. The outer capsid VP5 and VP2 are involved in cell attachment and viral entry [9,10]. VP2 is the main target of neutralizing antibodies and determines the serotype. Non-structural proteins participate in viral replication [11], protein synthesis [12], assembly, maturation and export from the infected cells [13,14,15,16,17], and neutralization of cellular factors, notably those involved in the host antiviral response. Indeed, both NS3 and NS4 have major roles in counteracting the innate immune response, and in particular the type I interferon (IFN-α/β) [7,18,19,20,21]. Moreover, a recent study suggests that NS4 and NS3 could act together to enhance STAT1 interference [22].

NS4 is encoded from a second reading frame (+1) carried by segment 9, which also encodes for VP6. BTV-NS4 is a small protein, with 77–79 amino acids in length, that is conserved among orbiviruses (African horse sickness virus, Great Island virus, Epizootic hemorrhagic disease virus). Depending on the viral cycle and/or experimental conditions, NS4 has a variable intracellular localization. Mostly cytoplasmic in the early stages of infection, it is preferentially localized within the nucleoli 24 h post-infection due to a nucleolar addressing motif present at its N-terminal region [6,7]. The presence of NS4 confers an advantage for virus replication in cells pre-treated with IFN suggesting that NS4 is an antagonist of the IFN-α/β response and a key determinant of the BTV virulence [7,19]. This replicative advantage has also been observed in the African horse sickness virus [23,24]. Remarkably, the NS4 protein appears to block promoters other than those related to the IFN-α/β response, suggesting a more general shutoff of the host gene expression [19].

Despite the fact that BTV has been used extensively as a model to study the *Orbivirus* replication cycle and structural biology, only a few interactions at the molecular level between viral and cellular proteins have been described so far. Moreover, classical BTV serotypes are very different from the atypical BTVs as regards their pathogenicity, virulence and capacity to infect and spread to mammal(s) host(s). Thus, we still have little understanding of the molecular determinants of their virulence and the evolutionary and molecular processes that affect changes in the host range. For example, BTV8 is virulent in sheep but can also induce clinical signs in cattle [25,26] whereas BTV27 infects exclusively goats without causing any symptoms [27].

In our study, we have used the yeast two-hybrid (Y2H) system to search direct cellular partners of the BTV-NS4 virulence factors encoded by BTV8 and BTV27. This led to the identification of nine new targets of BTV-NS4, including Wilms’ tumor 1-associated protein (WTAP). WTAP is involved in the N6-methyladenosine (m6A) methylation of RNA, one of the most ubiquitous and abundant modifications in both coding and noncoding RNAs [28,29]. Interestingly, NS4 protein encoded by BTV8, BTV1, BTV4 and BTV25 interacted with WTAP whereas BTV27-NS4 and BTV30-NS4 were unable to achieve this. The use of a systemic deletion-based mapping procedure as well as a BTV27/8-NS4 chimeric protein led us to define the WTAP and NS4 binding domains. Finally, we provided unprecedented evidence for the crucial role of WTAP in BTV replication.

## 2. Materials and Methods

### 2.1. Cell Lines and Viral Infections

HEK-293T cells were maintained in Dulbecco’s modified Eagle’s medium (DMEM; Gibco-Invitrogen) containing 10% fetal bovine serum, 100 units/mL penicillin, 1 mM sodium pyruvate and 100 μg/mL streptomycin at 37 °C and 5% CO_2_. Bovine umbilical cord endothelial cells (BUcEC, kindly provided by Dr. Anne-Claire Lagrée) were immortalized as previously described [30] and maintained in Opti-MEM (Gibco-Invitrogen) containing 5% fetal bovine serum and 0.4% gentamycin at 37 °C and 5% CO_2._ BTV8 strain (isolated in the French Ardennes in 2006 [31]) was amplified and titrated on BSR. BTV infection was analyzed at 24 h by western blotting using specific NS3 (kindly provided by Dr. Frederick Arnaud) [32] and VP5 antibodies (Mab 10AE12; Eurofins-Ingenasa). 

### 2.2. Plasmid DNA Constructs

NS4-encoding sequences from three French BTV strains (BTV8, 2006 [31]; BTV27, Corsica 2014 [27]; BTV4, Corsica 2016 [33]) and two BTV strains kindly provided by Dr. Martin Beer and Dr. Bernd Hoffmann (BTV25, Germany 2018 [34]; BTV30, Mongolia 2016 [3]) were amplified by two-step RT-PCR (Roche) from purified infected-cell RNAs. In contrast to others, BTV1-NS4 has been cloned from a plasmid (kindly provided by Dr. Piet A. van Rijn) containing segment 9 of a BTV1 strain generated by reverse genetics [35]. BTV-NS4 specific primers used for PCR were flanked with the Gateway^®^ cloning sites (5′-ggggacaactttgtacaaaaaagttggc-3’ and 5′-ggggacaactttgtacaagaaagttgg-3’). PCR products were cloned by in vitro recombination into pDONR207 (BP reaction; Invitrogen) and their sequences were verified. ORFs encoding bovine WTAP and Coiled-coil alpha-helical rod protein 1 (CCHCR1) were amplified from the MDBK cDNA library (see Yeast two-hybrid screening procedure), then cloned in pDONR207, and sequence verified. Goat WTAP was obtained by introducing six codon changes in the pDONR207-bovine WTAP construct by site-directed mutagenesis following the manufacturer’s instructions (QuikChange II site-directed mutagenesis, Agilent). The chimeric BTV27-NS4^22−55BTV8-NS4^ construction was synthesized and cloned into pDONR207 by GeneCust company (service delivery). ORF coding sequences were subsequently transferred by in vitro recombination from pDONR207 into different Gateway^®^-compatible destination vectors following the manufacturer’s recommendation (LR reaction, Invitrogen). 

### 2.3. Yeast Two-Hybrid (Y2H) Screening Procedure

Our Y2H protocol was largely inspired from the report of Vidalain et al. [36]. DNA sequences encoding for NS4 proteins were transferred by in vitro recombination (LR cloning reaction, Gateway^®^ technology, Invitrogen) from pDONR207 into the Y2H vector pDEST32 (Invitrogen) in order to be expressed in fusion downstream of Gal4 DNA-binding domain (Gal4-BD). Bait constructs were transformed into Y2H Gold yeast strain (Clontech) using a standard Lithium/Acetate procedure and selected on a synthetic medium lacking leucine (-L). Spontaneous transactivation of the *HIS3* reporter gene by BTV-NS4 proteins was determined on synthetic medium lacking histidine (-H) and supplemented with 3-amino-1,2,4-triazole (3-AT). For each BTV NS4 protein, we evaluated at 5mM the appropriate concentration of 3-AT preventing yeast growth in the absence of interacting prey protein. A mating strategy was used for screening the bovine cDNA library cloned in the Gal4-AD pDEST 22 vector (Invitrogen), previously transformed into the yeast strain Y187 (Clontech) and selected on a synthetic medium lacking tryptophan (-W). The bovine cDNA library is derived from the Madin-Darby bovine kidney (MDBK) cell line and its preparation was subcontracted (Life Technologies). For each screen, 20–50 million yeast diploids were produced and grown on selective medium -L, -W, -H supplemented with 5 mM of 3-AT. After 6 days of culture, [His+] colonies were picked and purified over 3 weeks by culture on selective medium to eliminate false-positives [37]. AD-cDNAs were amplified by PCR from zymolyase-treated yeast colonies using primers that hybridize within the pDEST 22 regions flanking cDNA inserts. PCR products were sequenced, and cellular interactors were identified by multiparallel BLAST analysis. 

### 2.4. Gap Repair (GR)

The PCR products corresponding to the previously identified cellular preys were also used to retest each interaction one by one in yeast following the gap-repair procedure [38]. Using the same standard Lithium/Acetate procedure as mentioned above, 10 ng of linearized pPC86 empty vector was co-transformed with 3 μL of PCR product to allow its recombinatorial repair in fresh Y2H Gold yeast cells expressing BD-fused BTV-NS4 proteins. Homologous recombination between the vector and the PCR product led to the reconstitution of AD-fused cDNA and growth on -L, -W, -H medium + 5mM of 3-AT was conditioned by physical interaction between BTV-NS4 and indicated cellular proteins.

To perform the refined mapping of NS4 and WTAP binding sites, we also used the GR procedure. First, both forward and reverse PCR primers were designed every 66 nucleotides along the BTV8-NS4 sequence, or every 150 nucleotides along the WTAP sequence and were fused to specific tails allowing recombination in the Gal4-BD or the Gal4-AD Y2H vector. The sequences of the specific tails in the Gal4-BD vector were 5′-GAAGAGAGTAGTAACAAAGGTCAAAGACAGTTGACTGTATCGTCGAGG-3′ and 5′-CCGCGGTGGCGGCCGTTACTTACTTAGAGCTCGACGTCTTACTTA-3′, and 5′-GATGAAGATACCCCACCAAACCCAAAAAAAGAGGGTGGGTCGAATCAA-3′ and 5′-CCGCGGTGGCGGCCGTTACTTACTTAGAGCTCGACGTCTTACTTA-3′ were used for the Gal4-AD Y2H vector. Matrix combinations of forward and reverse primers were used to amplify BTV8-NS4 and WTAP fragments by PCR. As described above, 10 ng of linearized pPC86 or pDEST32 empty vector was co-transformed with 3 μL of PCR product to achieve recombinatorial cloning by GR in yeast expressing BD-fused BTV8-NS4 or AD-WTAP. Interactions with BTV8-NS4 and WTAP were tested by plating yeast cells on -L, -W, -H medium and supplemented with 5 mM of 3-AT.

### 2.5. Co-Affinity Purification Experiments

To perform co-affinity purification experiments, ORFs encoding NS4 and WTAP or fragments, or CCHCR1 were transferred from pDONR207 to either pDEST27 (Invitrogen) or pCI-neo-3xFLAG expression vector to achieve GST and 3xFLAG fusion [39], respectively. HEK-293T cells were dispensed in each well of a 6-well plate (2 × 10^6^ cells), and 24 h later transfected (JetPRIME; Polyplus) with 500 ng of each plasmid DNA per well. Two days post-transfection, cells were collected in PBS and then incubated on ice in lysis buffer (20 mM MOPS-KOH pH 7.4, 120 mM of KCl, 0.5% Igepal, 2 mM β-Mercaptoethanol, supplemented with Complete Protease Inhibitor Cocktail (Roche)) for 20 min. Cell lysates were clarified by centrifugation at 14,000× *g* for 30 min. For pull-down analysis, protein extracts were incubated for 2 h at 4 °C on a spinning wheel with 30 μL of glutathione-sepharose beads (Amersham Biosciences) to purify GST-tagged proteins. Beads were then washed 3 times for 5 min with ice-cold lysis buffer and on a spinning wheel and, proteins were recovered by boiling in denaturing loading buffer (Invitrogen). 

### 2.6. Western Blot Analysis

Purified complexes and protein extracts were boiled at 95 °C for 5 min and resolved by SDS-polyacrylamide gel electrophoresis (SDS-PAGE) on 4–12% NuPAGE Bis–Tris gels with BOLT MES SDS running buffer and transferred to a nitrocellulose membrane in wet conditions (Invitrogen). GST and 3xFLAG-tagged proteins were detected with a rabbit polyclonal anti-GST antibody (1:2500, Sigma-Aldrich) and a mouse monoclonal HRP-conjugated anti-FLAG antibody (M2 1:10,000; Sigma-Aldrich), respectively. Specific antibodies were used to detect endogenous WTAP (A301-436A 1:1000) and actine (A 3853 1:2500) and purchased from Bethyl Laboratories and Sigma, respectively. Secondary anti-rabbit and anti-mouse HRP-conjugated antibodies were purchased from Invitrogen (1:5000). Nitrocellulose membrane was then incubated with a peroxidase substrate (Clarity™ Western ECL Substrate, Biorad) and visualized with the ChemiDoc MP Imaging System (Biorad).

### 2.7. WTAP Silencing

BUcEC were transfected with siRNAs using JetPRIME (Polyplus) according to the manufacturer’s instructions. Non-specific control and WTAP specific siRNAs were purchased from GeneCust and used at a final concentration of 25 nM. For WTAP specific siRNAs, the sequences used were 5′-CCAGCGAUCAACUUGUUAUTT-3′/5′-AUAACAAGUUGAUCGCUGGTT-3′ while 5′-UUCUCCGAACGUGUCACGUTT-3′/5′-ACGUGACACGUUCGGAGAATT-3′ were used as a negative control and noted as siCTR. One day later, BUcEC were infected with BTV8 (MOI = 0.01) for 24 h.

### 2.8. RT-qPCR in BUcEC

BUcEC were directly lysed in 0.35 mL of RLT buffer (QIAGEN) containing 1% 2-Mercaptoethanol. Total RNA was extracted using RNeasy columns (QIAGEN) according to the manufacturer’s instructions. RT-qPCR was performed using Quantifast SYBR Green RT-PCR Kit (Qiagen) with 50 ng of total RNA and experimental or control primers. The sequences of experimental primers targeting *WTAP* and the BTV segment 1 were 5′-CTACTCAGATCCAGTACCTCAAGCAA-3′ and 5′-CATTTTGGGCTTGTTCCAGTTT-3′, and 5′-GTTCCGCGCTAAAAACGAGA-3′ and 5′-CCCTGGTGGAATGGTGAATC-3′, respectively. Target transcripts were normalized to the control housekeeping gene, *GAPDH*, and the sequences used were 5′-GGTCGGAGTGAACGGATTCG-3′ and 5′-ACTCCACCACATACTCAGCA-3′. Reactions were performed using the LightCycler LC96 (Roche) and expression levels were analyzed using LightCycler 96 SW1.1 (Roche). The expression of *WTAP* and the BTV segment 1 were first normalized to the expression of *GAPDH*, then to the target gene expression of siCTR samples to calculate 2^−Δ(ΔCT)^ relative expression values, as specified in the figure legend.

### 2.9. Statistical Analyses

Statistical significance was assessed using the statistical package in GraphPad Prism, version 9.3.1. *p*-values are a result of unpaired two-tailed Student’s *T* test. Differences were considered to be significant if *p*-value < 0.05 (*) or < 0.005 (**) or < 0.0005 (***).

## 3. Results

### 3.1. Mapping Cellular Interactors of the BTV-NS4 Protein

**Figure 1:**
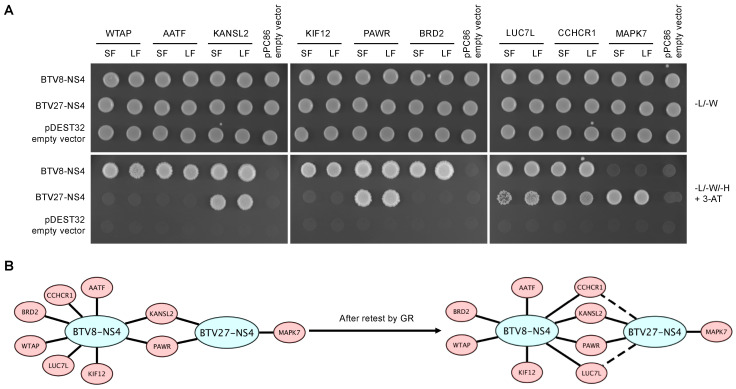
Validation of interactions between BTV-NS4 and cellular proteins. (**A**) Yeast cells expressing Gal4-BD alone or fused to either BTV8-NS4 or BTV27-NS4 were co-transformed with PCR products encoding cellular preys in the presence of linearized pPC86 vector to achieve recombinatorial cloning by gap-repair (GR). For each cellular prey, the shortest (SF) and longest (LF) fragments, identified in our Y2H screens, were used to retest the bait-prey combinations. -L/-W: synthetic culture medium depleted of leucine and tryptophan. -L/-W/-H + 3-AT: synthetic culture medium depleted of leucine, tryptophan, histidine + 5 mM of 3-amino-triazole to test the interaction-dependent transactivation of HIS3 reporter gene. (**B**) BTV8 and BTV27-NS4 proteins are represented in blue and pink nodes indicate bovine (*Bos taurus*) proteins identified in our screens. Solid lines denote interactions identified in our Y2H screens and confirmed by GR whereas dashed lines denote interactions only identified by GR.

To identify cellular partners of BTV-NS4, a cDNA library originating from cattle was screened by Y2H using full-length NS4 viral proteins from two serotypes (BTV8 and BTV27) as baits. Each screen was performed by yeast mating to obtain a minimum of 30.10^6^ diploids, a number that corresponds to 10-times the complexity of our cDNA library. A total of 288 positive [His+] yeast colonies were recovered from these two screens (206 and 82 clones for BTV8-NS4 and BTV27-NS4, respectively) and cellular prey proteins were identified by cDNA amplification, sequencing and multi-parallel BLAST analysis. Only interactions supported by a minimum of 3 independent yeast colonies were conserved to build the high-confidence interaction matrix displayed. Indeed, it has been shown that interactions identified 3 or more times in a Y2H screening approach can be validated at more than 80% by the affinity co-purification [40]. This represents a total of 11 interactions (8 for BTV8-NS4 and 3 for BTV27-NS4) corresponding to 9 distinct cellular proteins (Table 1). From this dataset, each cellular interactor was retested against BTV8-NS4 and BTV27-NS4 by transforming the PCR products (two for each cellular prey) in the presence of linearized pPC86 vector to achieve recombinatorial cloning by gap-repair into fresh yeasts containing the viral baits (Figure 1A). As expected, both BTV8 and BTV27-NS4 were able to interact with PAWR and KANSL2 but we also found LUC7L and CCHCR1 as common interactors of BTV-NS4. However, we confirmed that WTAP, AATF, KIF12, and BRD2 were serotype 8-specific but MAPK7 was unique to BTV27-NS4 (Figure 1B).

### 3.2. NS4 Interaction with WTAP Is Only Confirmed with BTV8-NS4

**Figure 2:**
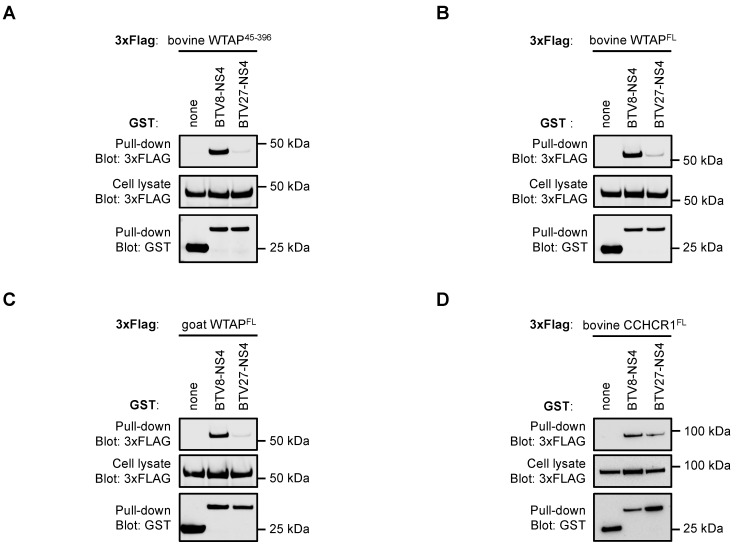
Only BTV8-NS4 interacts with WTAP. HEK-293T cells were transfected with expression vectors encoding GST alone or fused to the NS4 protein and tested for the interaction with either WTAP or CCHCR1. To achieve this goal, cells were co-transfected with a pCiNeo-3xFLAG expression vector encoding the fragment of bovine WTAP starting from position 45 (WTAP^45−396^, **A**), the full-length bovine WTAP (**B**), the full-length goat WTAP (**C**) or the full-length bovine CCHCR1 (**D**). Total cell lysates were prepared 48 h post-transfection (cell lysate; middle panel), and co-purifications of indicated cellular proteins were assayed by pull-down using glutathione-sepharose beads (pull-down; upper panel). GST-tagged NS4 were detected by immunoblotting using anti-GST antibody (pull-down; lower panel), while indicated cellular proteins were detected with an anti-3xFLAG antibody. Sizes are shown in kilodaltons (kDa).

Among the nine cellular interactors of BTV-NS4, WTAP has been identified 34 times corresponding to the highest number of hits in our Y2H screens using BTV-NS4 as bait. Moreover, the serotype 8-specific binding to WTAP is very interesting and suggests a new function for BTV-NS4 that we decided to investigate. In order to validate the interaction with WTAP, GST-tagged BTV-NS4 proteins were co-expressed with 3xFLAG-tagged bovine WTAP^45−396^ in HEK-293T cells, and total lysates were purified 48h later with glutathion-sepharose beads. WTAP^45−396^ constitutes the longest fragment of WTAP that was largely identified in our Y2H screen using BTV8-NS4 as bait. As shown in Figure 2A, 3xFLAG WTAP^45−396^ co-purified with BTV8-NS4. Interestingly, BTV27-NS4 failed to do so, thereby confirming the serotype 8-specificity of this interaction already observed in Y2H. As expected, full-length bovine WTAP also interacted only with BTV8-NS4 (Figure 2B). Since BTV27 principally infects goats, we tested if its NS4 protein was able to bind a goat form of WTAP. Again, only BTV8-NS4 copurified with goat WTAP (Figure 2C). In contrast, NS4 proteins from BTV8 and BTV27 had similar binding capacities for CCHCR1 (Figure 2D) suggesting that BTV27-NS4 was still functional. Altogether, these results confirmed data obtained from our Y2H screens where only BTV8-NS4 interacts with WTAP. 

### 3.3. NS4 Interaction with WTAP Does Not Discriminate Classical from Atypical BTV Serotypes

**Figure 3:**
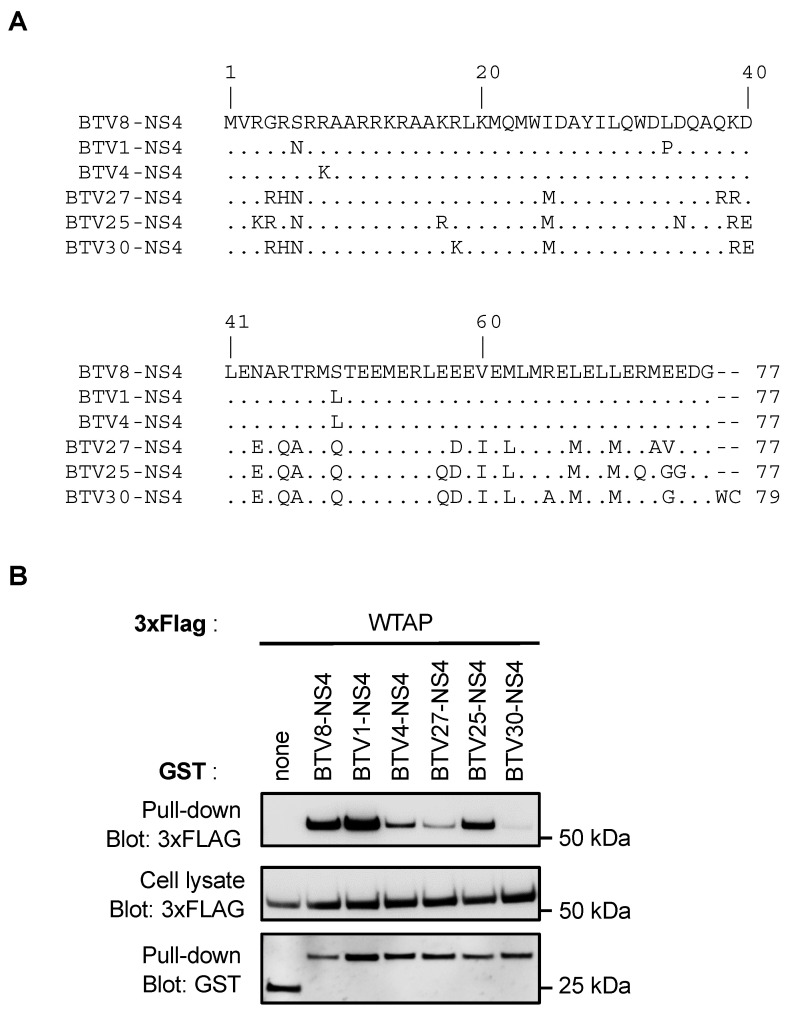
Comparative analysis of NS4 proteins from different BTV serotypes. (**A**) NS4 protein sequences from BTV8, BTV1, BTV4, BTV27, BTV25 and BTV30 were aligned with Clustal Omega version 1.2.3. (**B**) HEK-293T cells were transfected with expression vectors encoding GST alone or fused to BTV8-NS4, BTV1-NS4, BTV4-NS4, BTV27-NS4, BTV25-NS4 or BTV30-NS4 as indicated and tested for interaction with 3xFLAG fused to the full-length bovine WTAP. Total cell lysates were prepared at 48 h post-transfection (cell lysate; middle panel), and co-purifications of WTAP were assayed by pulldown using glutathione-Sepharose beads (pulldown; upper panel). Sizes are shown in kilodaltons (kDa).

We compared the amino acid (AA) sequences of NS4 proteins from three classical serotypes (BTV8, BTV1 and BTV4) and three atypical serotypes (BTV27, BTV25 and BTV30). As shown in Figure 3A, NS4 proteins from BTV8, BTV1 and BTV4 were highly conserved, with only four AA substitutions (AA6, AA8, AA34 and AA49). Similarly, the AA NS4 sequences were also very conserved between the atypical serotypes. However, NS4 proteins from BTV27, BTV25 and BTV30 were clearly distinct from BTV8-NS4 in several AA positions. This led us to address the question of the specificity of the WTAP interaction between these two groups of serotypes. Thus, GST-tagged NS4 from BTV8, −1, −4, −27, −25 and −30 were expressed in HEK-293T cells and purified 48 h later with glutathione-Sepharose beads. NS4 proteins from BTV8, −1 and −4 interacted with WTAP even if the interaction with BTV4-NS4 was reduced compared to those of the other NS4 proteins (Figure 3B). BTV27-NS4 and BTV30-NS4 failed to interact with WTAP whereas BTV25-NS4 had similar binding capacity for WTAP to that observed for BTV8-NS4 and BTV1-NS4. Therefore, the interaction between NS4 and WTAP could not be characteristic of the classical group of BTV serotypes.

### 3.4. Mapping of NS4 and WTAP Binding Sites

**Figure 4:**
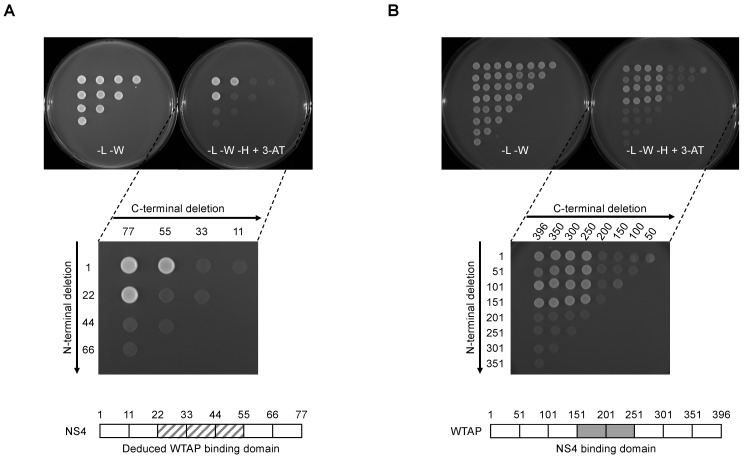
Yeast two-hybrid mapping of BTV-NS4 and WTAP binding regions. (**A**) Fragments of BTV8-NS4 were generated by PCR using a matrix combination of specific primers and introduced into Gal4-BD vector by gap-repair in yeast cells expressing WTAP fused to Gal4-AD. (**B**) In a similar approach, fragments of WTAP were tested for the interaction with BTV8-NS4. (**A**,**B**) -L -W: Selective medium lacking leucine and tryptophan to control the gap-repair and yeast transformation. -L, -W, -H + 3-AT: selective medium lacking leucine, tryptophan and histidine and supplemented with 5 mM of 3-amino-triazole. Vertical and horizontal axes indicate the first and the last amino acid residues of each fragment tested, respectively. The WTAP and NS4 binding regions that have been identified are represented in lower diagrams.

In order to characterize the NS4 binding interface on WTAP, we generated a full matrix of NS4 overlapping fragments by PCR and tested their ability to interact with WTAP in the Y2H system (Figure 4A). Both forward and reverse primers were designed every 66 nucleotides along the NS4 sequence (corresponding to every 22 amino acids) and fused to appropriate sequences to allow gap-repair recombination with linearized Gal4-BD Y2H vector. All possible combinations of forward and reverse primers were used to amplify NS4 fragments. Finally, corresponding PCR products with linearized Gal4-BD Y2H vector were transformed in a yeast strain expressing AD-fused WTAP, and growth on selective medium supplemented with 5 mM of 3-AT was used to detect potential interactions. Two fragments encompassing position AA1 to AA55 and AA22 to AA77 of NS4 were sufficient to bind WTAP (Figure 4A). These results suggest that the WTAP binding motif could be reduced to a minimal peptide of 34 AAs (BTV-NS4^22−55^) even if both N-terminal and C-terminal regions seem to be crucial for the interaction with WTAP. Using a similar approach, we then generated a set of WTAP fragments every 50 AAs (Figure 4B), allowing us to reduce the NS4 binding motif to a minimal domain encompassing AA151 to AA250 (WTAP^151−250^).

### 3.5. NS4^22−55^ Peptide Determines BTV-NS4 Binding Capacity for WTAP

**Figure 5:**
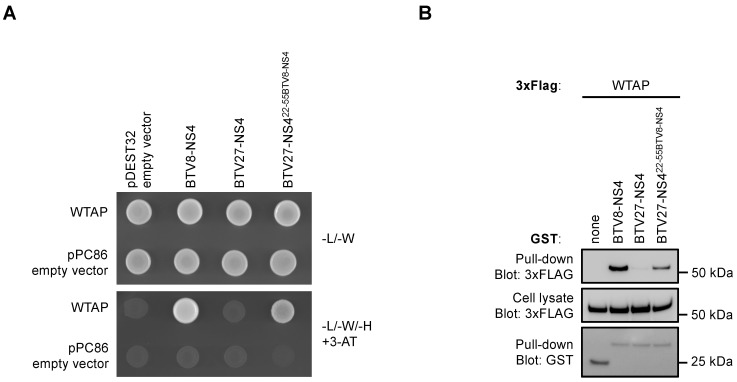
BTV-NS4 interaction with WTAP is mediated by a peptide encompassing residue 22 to 55. (**A**) Yeast cells expressing WTAP fused to Gal4-AD were transformed with a pDEST32 plasmid encoding BTV8-NS4, BTV27-NS4 or a chimeric BTV27-NS4^22−55BTV8-NS4^ protein, which consist of substitute BTV27-NS4 by BTV8-NS4 in the region encompassing residue 22 to 55. Synthetic culture medium depleted of leucine, tryptophan, histidine + 5 mM of 3-amino-triazole (-L/-W/-H + 3-AT) was used to test the interaction-dependent transactivation of HIS3 reporter gene. (**B**) HEK-293T cells were transfected with expression vectors encoding GST alone or fused to BTV8-NS4, BTV27-NS4 or BTV27-NS4^22−55BTV8-NS4^ and tested for interaction with 3xFLAG WTAP. Total cell lysates were prepared at 48 h post-transfection (cell lysate; middle panel), and co-purifications of WTAP were assayed by pulldown using glutathione-Sepharose beads (pulldown; upper panel). Sizes are shown in kilodaltons (kDa).

To confirm that the NS4^22−55^ peptide is responsible for WTAP binding, a chimeric BTV27-NS4^22−55BTV8-NS4^ protein, which consists to substitute BTV27-NS4 by BTV8-NS4 in the region encompassing residue 22 to 55, was artificially synthesized and tested for its potential binding to WTAP. Yeast cells expressing WTAP fused to Gal4-AD were transformed with Gal-BD-BTV8-NS4, -BTV27-NS4 or -BTV27-NS4^22−55BTV8-NS4^ and tested on a growth medium supplemented with 5 mM 3-AT. As shown in Figure 5A, BTV8-NS4 and BTV27-NS4^22−55BTV8-NS4^ but not BTV27-NS4 interacted with WTAP in Y2H. In a second approach, GST-tagged BTV8-NS4, BTV27-NS4 or BTV27-NS4^22−55BTV8-NS4^ were co-expressed in HEK-293T cells with expression vectors encoding for 3xFLAG-tagged WTAP. After 48 h, viral proteins were purified with glutathion-sepharose beads, and 3xFLAG-tagged WTAP was revealed by western-blot analysis. In agreement with data obtained by Y2H, only BTV8-NS4 and BTV27-NS4^22−55BTV8-NS4^ could bind WTAP even if the interaction was reduced with BTV27-NS4^22−55BTV8-NS4^ compared to BTV8-NS4 (Figure 5B). Altogether, these results demonstrated that the NS4^22−55^ peptide is involved in WTAP binding.

### 3.6. WTAP Is Essential for BTV Replication

**Figure 6:**
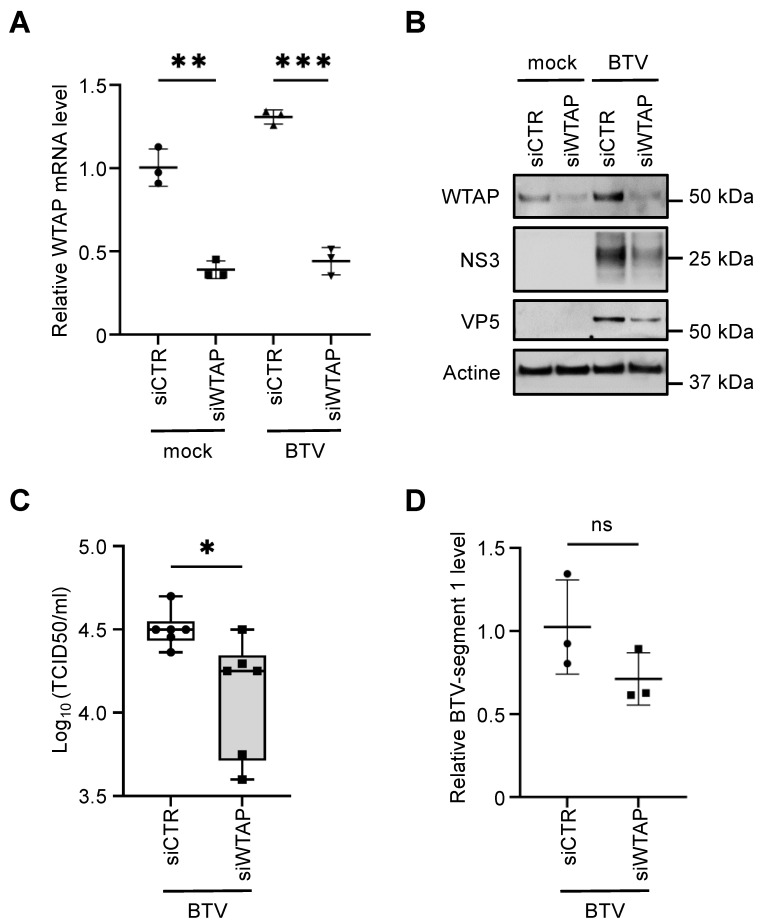
WTAP silencing alters BTV replication. (**A**–**D**) BUcEC were transfected with non-specific or a WTAP-specific siRNA. One day later, cells were infected with BTV8 (MOI = 0.01). After 24 h, relative WTAP mRNA and BTV-segment 1 levels were normalized to that of *GAPDH* (**A** and **D**, respectively). Cell lysates were analyzed by immunoblotting with antibodies against the indicated proteins (**B**) and the supernatants were titrated by determining the 50% tissue culture infective doses (TCID50)/mL (**C**). Experiments were achieved in triplicate (**A**,**B**,**D**) and with six individual samples for the viral titers (**C**). *, *p*-value (*p*) < 0.05; **, *p* < 0.005 and ***, *p* < 0.0005.

To test the physiological relevance of WTAP in BTV replication, we used a gene silencing approach targeting bovine WTAP in Bovine umbilical cord endothelial cells (BUcEC). BUcEC were transfected with WTAP-specific or non-specific control small interfering RNA (siRNA) before being infected with BTV8. First, the reduction of WTAP expression had been confirmed as assessed by RT-qPCR analyses (Figure 6A) and anti-WTAP immunoblotting (Figure 6B, upper panel). Then, WTAP silencing decreased the expression of BTV-NS3 and -VP5 (Figure 6B, middle panels) and exhibited significantly lower viral titers compared to the siRNA control (siCTR, Figure 6C). However, it should be noted that the level of BTV-segment 1 was not significantly reduced in cells pre-treated with siWTAP compared to the siCTR condition (Figure 6D) suggesting that WTAP could not have a role at the genome replication level. These results support a model where BTV-NS4 interaction with WTAP is important for BTV replication.

## 4. Discussion

In this report, we have performed a comparative yeast two-hybrid screening of the BTV-NS4 protein for identifying 11 interactions, including 8 for BTV8-NS4 and 3 for BTV27-NS4. From this dataset, each cellular interactor has been retested against BTV8-NS4 and BTV27-NS4 by gap-repair into fresh yeasts containing the viral baits. This step is essential to constitute a subset of interactions confidence. This allowed us to show that four cellular interactors, including WTAP, were specific to BTV8-NS4. WTAP forms a multiprotein complex with methyltransferase-like 3 protein (METTL3) and methyltransferase-like 14 protein (METTL14) to mediate N6-methyladenosine (m6A) methylation of RNAs that plays an important role in multiple stages of RNA life from its folding/structure, maturation, stability, to messenger RNAs (mRNA) splicing, export, translation, and decay. Therefore, m6A is involved in diverse biological processes and its dysregulation has been associated with a wide range of human diseases such as cancer or neurological and autoimmune disorders [41,42,43]. 

The m6A modification has also been found in viral RNA genomes and in viral mRNAs derived from both RNA and DNA viruses. The role of m6A in viral life cycle has been described for several viral families such as the *Flaviviridae*, *Orthomyxoviridae*, *Paramyxoviridae*, or the *Retroviridae* (see [44] for a review). However, and to the best of our knowledge, our model where BTV-NS4 interaction with WTAP is important for BTV replication, would constitute the first example of a proviral effect of m6A for a virus member of the *Reoviridae* family. The m6A modification is involved at multiple steps in the viral life cycle including the viral genome replication, the stability, splicing or translation of viral mRNAs, and the encapsidation of genomic RNAs [45]. In our report, we demonstrated that WTAP silencing led to reduced viral titers and expression of VP5 and NS3 viral proteins whereas our RT-qPCR data suggested WTAP could not have a role at the genome replication level. Further investigations are still required to evaluate if NS4, through its interaction with WTAP, acts directly on the viral life cycle or if it has a general proviral effect in BTV replication. 

In addition to acting directly on viruses, m6A has been reported to modulate both the innate and adaptive immune responses [46]. Innate immunity represents the first line of defense against viruses where the IFN-α/β constitute the principal mediators that stimulate the expression of hundreds of IFN-stimulated genes (ISGs). The IFN-α/β signaling pathway is initiated after the recognition of microbe-associated molecular patterns (MAMPs) by the pattern recognition receptors (PRRs), such as the RIG-I and MDA5 helicases that have been described as sensors of the BTV infection [47]. Evolving rapidly under the pressure of antiviral responses, viruses have developed escape strategies to the IFN-α/β pathway by preventing the recognition of MAMPs by the PRRs or blocking directly the IFN-α/β signaling pathway. It has recently been reported that m6A modification in the viral RNAs of the human metapneumovirus [48] and the hepatitis B and C viruses [49] can prevent virus-sensing by RIG-I and consequently inhibit the induction of the IFN-α/β signaling pathway and antiviral immunity. In the context of the vesicular stomatitis virus (VSV), Qiu et al. have demonstrated that m6A reshapes viral RNA duplex structure and consequently alters viral RNA sensing by RIG-I and MDA5 [50]. Moreover, m6A has also been shown to target IFNβ mRNA, enhancing its destabilization and reducing its production [51,52]. Interestingly, WTAP protein has also been shown to be degraded in cells infected by VSV, human herpes simplex virus 1 or Sendai virus. Its degradation was associated with a decrease of the m6A levels of IFN-regulatory factor 3 (IRF3) and interferon alpha/beta receptor subunit 1 (IFNAR1) mRNAs, leading to translational suppression of IRF3 and instability of IFNAR1 mRNA and, therefore, reducing the IFN-α/β response [53]. As BTV-NS4 is known to have an important role in counteracting the innate immunity, it is possible that NS4 protein, through its interaction with WTAP, could manipulate m6A modification to either prevent viral RNA sensing by RIG-I and MDA5 or regulate directly signaling molecules of the IFN-α/β signaling pathway. The latter hypothesis is more likely since the *IFN-β* gene was strongly upregulated in BTV NS4 deletion mutant (BTVΔNS4)-infected cells than in BTV wild type-infected cells whereas NS4 protein does not seem to prevent viral sensing by the PRRs [19]. In this report, authors have also suggested that the reduced level of IFN-β and other ISGs mRNAs could be due to the host protein shutoff induced by BTV [54,55,56] and, possibly by BTV-NS4. As the m6A modification can also act at both transcriptional and translational levels, our report provides instrumental data to further investigate if BTV-NS4 could modulate m6A to induce host protein synthesis shutdown and/or to target the IFN-β mRNA. 

Our findings support a model where BTV-NS4 interacts with WTAP to benefit its viral replication. To the best of our knowledge, this is the first report demonstrating WTAP as a target of a viral protein. WTAP interacts with METTL3 and METTL14, and is required for their localization into nuclear speckles and m6A methyltransferase activity [57]. Interestingly, another study has demonstrated that the METTL3 binding surface could be reduced to the first 150 AA of WTAP [58]. As we have identified the NS4 binding motif to a minimal domain encompassing AA151 to AA250 (WTAP^151−250^), we can reasonably suppose that METTL3 and BTV-NS4 would not compete for binding to WTAP during BTV infection. The WTAP binding motif has been identified (BTV-NS4^22−55^) and is carried by the predicted coiled-coil structure of BTV-NS4^27−77^ [6]. Although the AA sequence of BTV4-NS4 was almost identical to that of BTV8-NS4, with only one substitution in position 49 of the BTV-NS4^22−55^ region (Figure 3A), BTV4-NS4 was less efficient than BTV8-NS4 to interact with WTAP. On the other hand, BTV25-NS4 was clearly distinct from BTV8-NS4 in several AA positions but had a similar binding capacity for WTAP to that observed for BTV8-NS4. In agreement with these data, we have also shown that the interaction with WTAP is partially restored with a chimeric BTV27-NS4^22−55BTV8-NS4^ protein. Altogether, these results demonstrate that even if the WTAP binding motif is carried by the BTV-NS4^22−55^ region, both N-terminal and C-terminal regions are still important to reinforce the interaction with WTAP. 

To date, very few studies have described at the molecular level how viruses manipulate m6A modification for their own profit and inhibit the host’s antiviral response. Moreover, only a few interactions between viral and cellular components of m6A have been described so far. Most viruses, like BTV, infect a broad spectrum of hosts and some of them are even transmitted by an arthropod vector. Comparative molecular approaches at viral (between viruses) and host (vertebrate host versus arthropod vector) levels to investigate m6A modification of both cellular and viral RNAs will be an interesting avenue for future research.

## 5. Conclusions

Later on, our comparative Y2H approach will be extended to other viral proteins encoded by different strains of BTV but also by other orbiviruses. It would provide a better understanding of virus-host molecular interactions at multiple levels: viral (between orbiviruses) and host (mammals and arthropod vectors). Such interactions would determine the ability of a virus to cross species barriers (interspecies transmissions) and adapt to a new host. By comparing virus-host interactomes obtained from different BTV strains, we will be able to characterize protein interactions that would be either conserved throughout this virus or specific to highly virulent strains, explaining the virulence/pathogenesis associated with its infection. It would also be a great benefit to combine the Y2H system with other high-throughput standardized proteomic approaches to provide a better and global understanding of the cellular network of orbiviruses.

## Figures and Tables

**Table 1:** Summary of data from the Y2H screens. The table summarizes the number of interactions (hits) and cellular interactors that have been identified in our Y2H screens using BTV8 and BTV27-NS4 proteins as baits.

Protein Abreviation	BTV8	BTV27	Total Hits	UniProtKB ID	Protein Name
WTAP	34		34	F1MN80	Wilms’ tumor 1-associated protein
PAWR	19	25	44	F1MMF4	PRKC apoptosis WT1 regulator protein
KANSL2	18	8	26	Q2NL14	KAT8 regulatory NSL complex subunit 2
AATF	23		23	E1BDL9	Apoptosis antagonizing transcription factor
KIF12	18		18	E1BBY6	Kinesin family member 12
MAPK7		14	14	A5PKJ4	Mitogen-activated protein kinase 7
BRD2	7		7	Q32S26	Bromodomain containing 2
LUC7L	6		6	F1MMH3	LUC7 like
CCHCR1	5		5	F1N2Z6	Coiled-coil alpha-helical rod protein 1
Total hits	130	47	177		
Total interactors	8	3	9		

## Data Availability

All data are given in the Results section.
